# Microbe Profile: Acinetobacter baumannii: persist and resist

**DOI:** 10.1099/mic.0.001787

**Published:** 2026-10-13

**Authors:** Rubén de Dios, Ronan R. McCarthy

**Affiliations:** 1 National Biofilms Innovation Centre, School of Biological Sciences, Faculty of Environmental and Life Sciences, University of Southampton, University Road, Southampton, SO17 1BJ, UK

**Keywords:** *Acinetobacter baumannii*, antibiotic resistance, biofilm, desiccation

## Abstract

*Acinetobacter baumannii* is a Gram-negative opportunistic pathogen associated with a wide variety of healthcare-associated infections, including ventilator-associated pneumonia, urinary tract infections, bacteraemia and wound infections. It adopts a ‘persist and resist’ pathogenic strategy to establish infection in the host. It can form robust biofilms and survive in a desiccated state for months at a time, driving its persistence in the hospital-built environment. It also has a remarkable capacity to rapidly develop antimicrobial resistance, with >90% of contemporary clinical isolates being multidrug-resistant. This combination of pathogenic traits has caused *A. baumannii* to emerge as a major clinical challenge.

## Taxonomy

Domain: *Bacteria*; Kingdom: *Pseudomonadati*; Phylum: *Pseudomonadota*; Class: *Gammaproteobacteria*; Order: *Moraxellales*; Family: *Moraxellaceae*; Genus: *Acinetobacter*; Species: *Acinetobacter baumannii*.

*A. baumannii* was first defined as a distinct species by Bouvet and Grimont in 1986. It was named after Paul Baumann in recognition of his studies on the *Acinetobacter* taxonomy.

## Properties

Biochemically, *A. baumannii* is a Gram-negative bacterium, strictly aerobic, prototrophic, non-fermentative and oxidase-negative. It is a coccobacillus of 1.0–2.5 µm size, although it becomes spherical in stationary phase. It grows at a wide range of temperatures (22–45 °C) with the optimum around 37 °C. It has a doubling time of approximately 20–25 min in lysogeny broth at 37 °C. In terms of metabolism, *A. baumannii* can use the Entner–Doudoroff and pentose phosphate pathways, the tricarboxylic acid cycle, the glyoxylate shunt and gluconeogenesis. It can use a wide range of carbon and energy sources, with sugars being non-preferential [1].

From a clinical perspective, *A. baumannii* is a nosocomial pathogen that accounts for 80% of human infections caused by *Acinetobacter* species. It typically causes ventilator-associated pneumonia, urinary tract infections, bacteraemia and wound infections. It distinctively encodes the carbapenemase OXA-51, which provides an intrinsic background resistance to the last-resort antibiotics carbapenems. Recent reports show that >90% of modern *A. baumannii* clinical isolates are multidrug-resistant. Carbapenem-resistant *A. baumannii* has been listed as a Critical Priority Pathogen by the World Health Organization since 2017. Its ability to colonize humans, animals and the environment emphasizes the need to adopt One Health approaches for *A. baumannii* study and surveillance. Transmission typically occurs through direct contact (contaminated surfaces, medical equipment), although airborne transmission has also been reported, which can be assisted through hitchhiking on fungal spores [2].

## Phylogeny

Two MLST schemes are used for comparative phylogenetic analyses of *A. baumannii* strains: the Pasteur and Oxford schemes. Both systems are based on the allele variation of two partially overlapping groups of seven conserved genes. Although the Oxford scheme (*groEL*, *gpi*, *gltA*, *gyrB*, *recA*, *rpoD* and *gdhB*) may provide better resolution, the Pasteur scheme (*groEL*, *fusA*, *gltA*, *pyrG*, *recA*, *rplB *and *rpoB*) is currently considered more accurate for clonal lineage classification and epidemiological studies.

Originally, 3 clonal lineages of *A. baumannii* were defined across different countries in Europe, which were designated as International Clones (ICs) 1, 2 and 3 [Pasteur sequence types (STs) 1, 2 and 3, respectively]. However, the number of ICs has progressively expanded to 12. The most prevalent lineage worldwide is IC2, whereas IC3 is now uncommon. Other globally spread lineages include IC1 and IC4–8 (ST15, ST79, ST78, ST25, ST10, respectively). IC9–12 are the most recently emerged clones and belong to ST85, ST33, ST164 and ST158, respectively.

Although the evolutionary origin of *A. baumannii* remains unclear, recent studies indicate an early radiation of the species linked to human activity, which dates back to the early Neolithic period [2]. Recent evidence also suggests transient *A. baumannii* asymptomatic gut carriage in infants among the community, using amino acids to persist in this environment [1]. In this context, it is tempting to hypothesize that the parallel evolution between *A. baumannii* and humans has contributed to its recalcitrant pathogenic strategy.

## Genome

*A. baumannii* chromosomes are ~4 Mb circular DNA molecules with 38–40 mol% G+C content. ATCC 17978, AYE and ACICU were the first genomes sequenced of this pathogen in the early 2000s. As of today, there are over 40,000 *A*. *baumannii* genome records on GenBank. ATCC 17978, isolated in the 1950s before the antimicrobial resistance crisis, is considered the reference *A. baumannii* strain, although it lacks many of the genomic features associated with contemporary clinical isolates and emergent lineages. This lack of comparability is prompting *A. baumannii* researchers to shift to more modern models for experimental studies, such as ACICU (IC2), AB5075 (IC1), UPAB1 (IC7) or LAC-4 (IC8).

One of the most important features of *A. baumannii* is its highly dynamic genome. Transposition events, recombination, conjugation and natural transformation are crucial for rearranging endogenous genetic material and acquiring and stabilizing exogenous DNA. This level of genome plasticity enables rapid evolution and underpins the notoriously challenging antimicrobial resistance profile of this pathogen. The search for systems modulating the acquisition of mobile genetic elements revealed two mutually exclusive phylogenetic clusters: a group of strains encoding methylation-based restriction–modification systems and a separate group, mainly comprising the IC2 lineage, encoding an Ssp-like phage defence system that introduces DNA phosphorothioate modifications [3]. This has been hypothesized as a means to modulate the mobile genetic element profile across *A. baumannii* lineages. Linking back to phylogenetics, this intrinsic genomic variability has prompted the preferential use of pan-genomics, considering both core and accessory genomes, to understand short-term *A. baumannii* clonal expansion and evolution, for example, in the context of hospital outbreaks.

## Key features and discoveries

One of the most prominent traits of this pathogen is the ability to tolerate desiccation, i.e. surviving in the absence of water, for prolonged periods of time, with the most recalcitrant isolates enduring for months [4]. Combined with its ability to resist disinfectants, this is especially concerning in hospital settings, as modern *A. baumannii* isolates maintain and even increase their pathogenic potential even after resuscitation. The modulation of protein aggregation and the protective hydrophilins DtpA and DtpB are key to maintaining viability in low-humidity conditions [4]. Recently, desiccation tolerance has been linked to entry into the viable but non-culturable (VBNC) state. Although it has been recently described that intracellular levels of phenylacetic acid mediate this transition [5] and that divalent cations are key drivers of resuscitation, the molecular mechanisms governing this behaviour are yet to be fully elucidated.

Rather than LPS, *A. baumannii* displays a highly variable lipooligosaccharide (LOS) on its outer membrane, an LPS analogue lacking the O-antigen (Fig. 1). This structure is essential for maintaining permeability and rigidity of the outer membrane, but it acts as a main target for last-line cationic peptide antibiotics, such as colistin. However, under selective pressure, *A. baumannii* can acquire mutations in the LOS biogenesis pathway, developing resistance to these antibiotics and producing LOS-deficient cells. Unlike most Gram-negative bacteria, *A. baumannii* LOS-deficient cells remain viable due to the envelope structural support provided by peptidoglycan [6], whilst suffering a significant fitness cost *in vitro* and *in vivo*.

**Fig. 1.**
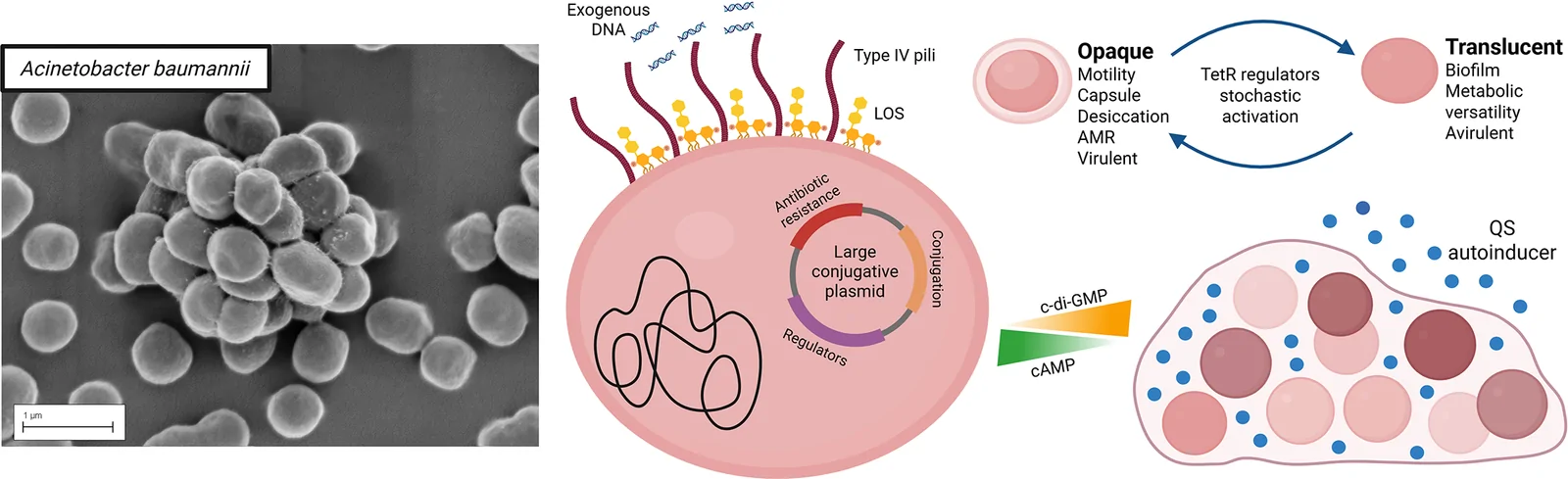
*A. baumannii* virulence, resistance and persistence determinants. Created with BioRender.

*A. baumannii* produces robust biofilms, which protect it from environmental stresses, including antibiotic treatments and the host immune system. The genes encoding the poly-*N*-acetylglucosamine biosynthesis machinery (*pga* operon)*,* the biofilm-associated proteins (*bap*) and different types of chaperone-usher pili (*csu*, *cup1*/*abp1* and *cup2*/*abp2*) are essential for seeding and developing the biofilm structure. It features a quorum sensing (QS) system characterized by the autoinducer synthase AbaI, which synthesizes N-(3-hydroxydodecanoyl)-l-homoserine lactone (3-OH-C12-HSL), and the cognate response regulator AbaR. This QS system has been shown to influence a range of phenotypes, including biofilm and surface-associated motility. Recent work has revealed that the second messengers cyclic AMP (cAMP), through the CavA-Vfr pathway, and cyclic di-GMP (c-di-GMP), through an intricate network of c-di-GMP cyclases and phosphodiesterases that diverges between different lineages, antagonistically control virulence and determine environmental adaptability and host colonization [7, 8]. Importantly, these two second messengers regulate the expression of the *pil* and *com* genes in an opposite manner to the biofilm formation determinants. These genes encode the Type IV Pili machinery and associated proteins that mediate motility and natural transformation, a cornerstone of antibiotic resistance gene acquisition in this pathogen.

Many strains of *A. baumannii* show a bi-stable phenotype, called phase variation, that defines their global behaviour [9]. The switch between the two phases, known as opaque and translucent, is controlled by the stochastic activation of a panel of TetR regulators modulated by different cues, including cell density (independently of QS) and CsrA activation. The opaque and translucent states can be distinguished on solid media under oblique indirect illumination, with colonies showing two levels of opacity. Colonies in each state are associated with distinct phenotypes: opaque colonies generally show increased virulence, capsule production, motility and desiccation tolerance, whereas translucent cells show enhanced biofilm formation and greater metabolic versatility.

In terms of interplay with the host, a relevant feature determining this interaction is the ability of some modern *A. baumannii* strains to replicate inside macrophage vacuoles, using them as a transient reservoir during infection [10]. This intracellular lifestyle can contribute to within-host dissemination, immune evasion and antibiotic treatment failure particularly with respect to urinary tract-associated infections. Importantly, the cross-talk between large conjugative plasmids and the chromosome plays a role in the modulation of this process.

## Open questions

- What makes clinical isolates of *A. baumannii* pathogenic in comparison to environmental isolates and other *Acinetobacter* species? What is the origin of the epidemic clones and their natural habitat?
- What are the molecular drivers of desiccation tolerance and the regulators that mediate entrance into VBNC state and resuscitation?
- What are the best targets for developing novel anti-virulence and anti-biofilm therapeutics? And for vaccine development?
- What is the role of plasmids and other mobile genetic elements in the rapid emergence of MDR, XDR and PDR isolates? What defines their adaptation and interplay with *A. baumannii* as a host?
- What are the determinants of *A. baumannii* virulence and its interaction with the host?

## Abbreviations

c-di-GMP: cyclic di-GMP
ICs: International Clone
LOS: lipooligosaccharide
QS: quorum sensing
STs: Sequence Type
VBNC: viable but non-culturable
